# Maturation and Function of the Intercalated Disc: Report of Two Pediatric Cases Focusing on Cardiac Development and Myocardial Hyperplasia

**DOI:** 10.3390/jcdd10080354

**Published:** 2023-08-19

**Authors:** Willem B. van Ham, Esmeralda E. M. Meijboom, Merel L. Ligtermoet, Peter G. J. Nikkels, Toon A. B. van Veen

**Affiliations:** 1 Department of Medical Physiology, University Medical Center Utrecht, 3584 CX Utrecht, The Netherlands; 2 Department of Pathology, University Medical Center Utrecht, 3584 CX Utrecht, The Netherlands

**Keywords:** intercalated disc, myocardial hyperplasia, sudden death

## Abstract

The development of the normal human heart, ranging from gestational age to the mature adult heart, relies on a very delicate and timely orchestrated order of processes. One of the most striking alterations in time is the gradual extinction of the ability for cardiomyocytes to proliferate. Once passing this event, cardiomyocytes grow and increase in contractile strength by means of physiological hypertrophy. This process, importantly, seems to depend on an adequate development of electromechanical coupling that is achieved by the appropriate formation of the intercellular junction named the intercalated disc (ICD). In this report, we describe two sudden death cases of young and apparently healthy-born individuals without external abnormalities compared to an age-matched control. Histological examination, including the comparison with the age-matched and histology-matched controls, showed a disturbed formation of the protein machinery composing the electromechanical junctions at the ICD and an increased nuclei count for both patients. As a cause or consequence, cardiomyocytes in both sudden death cases showed signs of a delayed developmental stage, presumably resulting in an exaggerated degree of hyperplasia.

## 1. Introduction

The interconnection of cardiomyocytes in the adult human heart is the basis for myocardial stability and function, resulting in the synchronous electromechanical activation of the heart. The area at which the cardiomyocytes are connected in a longitudinal orientation is named the intercalated disk (ICD). It consists of a plethora of components that are structured in a sophisticated manner, thereby forming a network of neighboring proteins and the cytoskeleton of the cell [1]. The intricate complex of junctional proteins, ion channels, and connexin channels is also defined as the area composita. Proteins such as N-cadherin (N-CAD), β-catenin, and plakoglobin (JUP) play an important role in the mechanical coupling of the cardiomyocytes. As cardiac workload increases during fetal and neonatal development [2], mechanical reliability becomes imperative. The absence of N-CAD during embryogenesis is therefore also resulting in compromise of the myocardium, delayed development, and embryonically lethal [3,4]. During cardiomyocyte maturation, these ICD proteins, as well as gap junction proteins (connexins), are initially dispersed across the entire cell. Over time, they localize on the lateral sites of the cell and finally on the longitudinal ends [5,6]. Altered expression or localization of these proteins has been connected to cardiomyopathies and is linked to an increased incidence of arrhythmias [7,8]. This highlights the significant relationship between mechanical and electrophysiological integrity.

One of the first organs to develop and function during embryogenesis is the heart. Throughout the subsequent phases of embryonic and fetal cardiac development, cells proliferate and migrate to form a heart tube, and due to the looping process of the tube over a timespan of around 7 weeks, they form a four-chamber system [2]. In mammalian hearts, this proliferative capacity rapidly diminishes around birth, upon which the cardiomyocytes undergo physiological hypertrophy to increase cell size, protein expression, and support sarcomere organization [9]. In the human heart, hyperplasia also ceases gradually postnatally, leading to a negligible role in determining heart size after the age of 20 [10,11]. This process leads to terminal differentiation of the cardiomyocytes, which coincides with a general arrest of the capacity for cell division. Instead, cardiomyocytes undergo an alternate cell cycle, named endomitosis, during which the genome is replicated without finishing karyokinesis during mitosis [12,13]. This results in polyploid nuclei in cardiomyocytes, which is presumed to facilitate increased protein production, growth, and ICD stabilization in the hypertrophic cells. Interestingly, when karyokinesis does ensue but subsequent cytokinesis fails, cardiomyocytes end up multinucleated. This is mainly observed in animals like mice, dogs, and pigs, while human cardiomyocytes are predominantly mononucleated [10,14]. During genome replication, the chromosomes are protected by the Ki67 protein, which can therefore be used as a marker for cells that are or have relatively recently been proliferating [15]. The shift from proliferation towards hypertrophy is a complex and not fully understood mechanism, but the process of polyploidy is thought to be connected. Furthermore, the ICD components N-CAD and β-catenin have also been associated with playing a role in this transition [16,17].

Here we report an observational study of two pediatric patients who died of asphyxia without any known cause. Both cases presented during autopsy with enlarged hearts and an indication of malformed ICD structures. We performed an immunohistological study of cardiac tissue obtained from those two patients, in which we identified immaturely formed ICD structures. Additionally, patient material indicated persistent hyperplasia of the cardiomyocytes, which presumably could be responsible for insufficient contractile function and enhanced susceptibility to cardiac arrhythmias.

## 2. Materials and Methods

Cardiac specimens of both patients (S and T, 4 and 6 weeks postnatal, respectively), an age-matched control (R, 5 weeks postnatal), and a histology-matched control (Q, 39 weeks in utero) were acquired via the biobank from the Department of Pathology of the University Medical Center Utrecht, the Netherlands. Both patients and the 5-week age-matched control were full term infants. Patient material was cryofrozen in liquid nitrogen. Tissues were then sectioned onto glass slides and post-fixed in 4% paraformaldehyde prior to immunohistology. Tissues were washed once with PBST (0.2% tween-20 in PBS) and once with TBST (20 mM tris, 150 mM NaCl, and 0.2% tween-20 in PBS, pH = 8.0). Triton X-100 (0.2% in PBS) was used to permeabilize the cell membranes, followed by a brief wash with PBST. Samples were then blocked with BSA (4% BSA in PBS) for 60 min at room temperature. Incubation of the first antibodies was done overnight at 4 °C in 2% BSA in PBS. The next day, tissues were washed five times for 5 min with PBST, and secondary antibody incubation was again performed in 2% BSA in PBS for 120 min at room temperature. Afterwards, tissues were washed two times for 5 min with TNT (100 mM tris, 150 mM NaCl, and 0.1% tween-20 in ddH_2_O, pH = 8.0), as well as three times with PBST. Finally, slices were dried, covered, and stored at −20 °C. Labeling was performed against N-CAD (Sigma, St. Louis, MO, USA, C3678, 1:800), JUP (Sigma, P8087, 1:500), Ki67 (Invitrogen, Carlsbad, USA, PA519462, 1:500), α-actinin (Sigma, A7811, 1:1000), β-catenin (BD Transduction, Franklin Lakes, NJ, USA, 610154), and DAPI (Life Technologies, Carlsbad, CA, USA, D1306, 1:100) as counterstain to visualize the nuclei. Images were captured on a Nikon Eclipse Ti2 microscope with 40× and 60× objectives, and MetaMorph software (v.7). In total, 6–8 images taken from 4 independent tissue slice stainings per experiment were processed using ImageJ with the “cell counter” and “quickfigures” plugins. GraphPad Prism (v.10) was used for data visualization and to statistically compare nuclei counts with a One-way ANOVA with Tukey’s test for multiple comparisons.

## 3. Case History

Patient S was a 4-week-old male without a personal or familial medical history. The day prior to his death, the patient experienced tachypnea and was admitted to the ER, where resuscitation was later started due to apnea without shockable heart rhythm. The infant had normal proportions, weighing 4060 g and a length of 57.5 cm. The heart was enlarged, weighing 42 g, with normal anatomy and the absence of dilation or inflammation. Patient T was a 6-week-old male with no previous medical history. The patient suffered from an irregular breathing pattern, which culminated in the need for resuscitation, during which asystole was noticed. Again, the infant had no external abnormalities, weighing 5600 g and a length of 57 cm. The heart was also enlarged, weighing 35.6 g, without inflammation or deformations. No abnormalities were identified in the other organs of either patient.

The histology-matched control (control Q) was a male fetus of 39 weeks that died in utero due to late-stage defective placental maturation. Aside from signs of recent hypoxia, such as mild aspiration of amniotic fluid and the absence of increased erythropoiesis, the fetus had no congenital abnormalities. The body weight was 3240 g with a length of 52 cm. No anatomical anomalies were identified, and the heart weight was 20.4 g. The age-matched control (control R) was a 5-week-old female patient with Pierre Robin sequence who suffered from severe cerebral ischemia during surgery and had treatment withheld based on a very poor prognosis. Beyond the classical signs of the Pierre Robin sequence, the infant had no other congenital anomalies and normal proportions, weighing 4400 g and having a length of 56 cm. Cardiac anatomy was normal, and the heart weight was 25.3 g.

**Figure 1:**
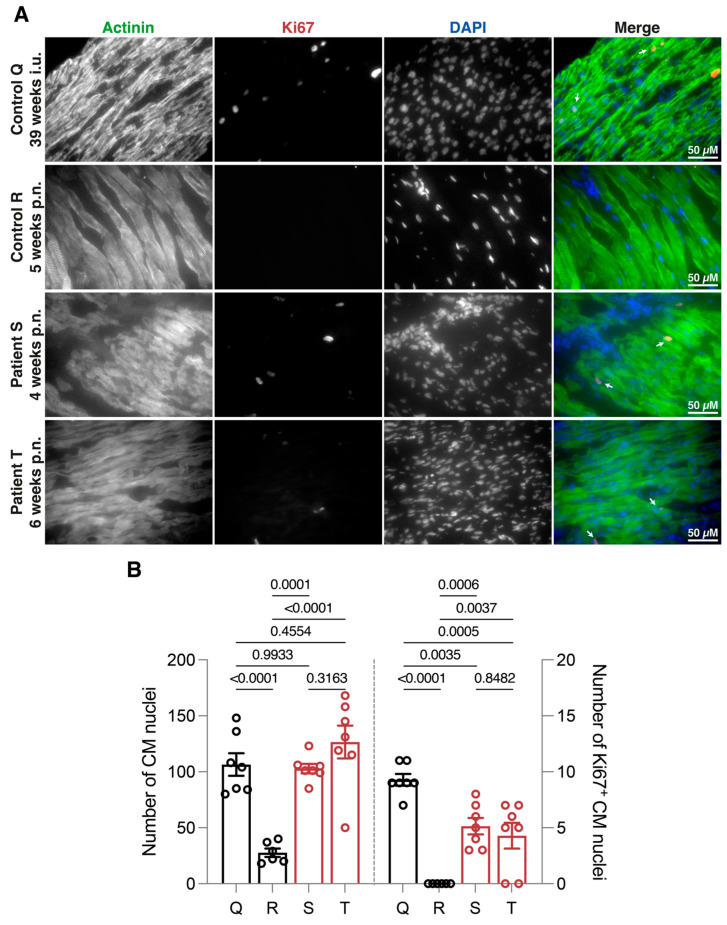
Hyperplasia and cardiomyocyte proliferation in pediatric patient tissues. (**A**) Immunohistochemical labeling of cardiomyocytes (α-actinin), proliferating cells (Ki67), and nuclei (DAPI), at 40×. (**B**) Comparison of two pediatric patients (patient S, 4 weeks postnatal; patient T, 6 weeks postnatal) to age-matched (control R, 5 weeks postnatal) and histology-matched (control Q, 39 weeks in utero) controls showed increased nuclei count and proliferation-positive nuclei (white arrows). CM—Cardiomyocyte. A total of 6–8 images were analyzed based on 4 independent tissue slice stainings per individual. The scale bar represents 50 µM.

Autopsy reports of both patients highlighted the similarities of enlarged hearts without dilation as well as an initial indication of malformation of the ICD structures. Immunohistological labeling of both hearts was performed to determine the nuclei count and the presence of the proliferation marker Ki67. These data were compared with control Q and control R, which both died without evidence of cardiac involvement. Cardiomyocyte-specific α-actinin signals indicated well-organized fibers of larger cardiomyocytes in tissue of control R without the presence of Ki67-positive immunolabeling (Figure 1A). In contrast, both patients showed an increased number of seemingly smaller-sized cardiomyocytes, highlighted by the combination of α-actinin-positive labeling and total nuclei counts (Figure 1B), suggesting the absence of hypertrophy during development. Furthermore, both hearts displayed an increased number of Ki67-positive nuclei, indicating increased cell proliferation, suggesting myocardial hyperplasia as the causative explanation for the enlarged but not dilated hearts. Interestingly, these labelings of patient material are similar to control Q, which indicates that the patients were in a hyperplastic phase comparable to 39 weeks of gestation.

**Figure 2:**
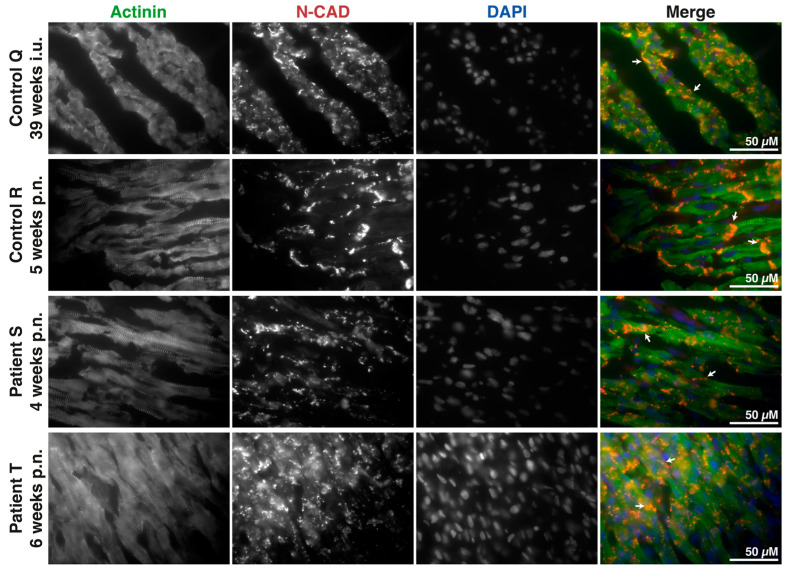
Immature intercalated disc maturation in pediatric patient tissues. Immunohistochemical labeling of cardiomyocytes (α-actinin), N-cadherin (N-CAD), and nuclei (DAPI) at 60×. Comparison of two pediatric patients (patient S, 4 weeks postnatal; patient T, 6 weeks postnatal) to age-matched (control R, 5 weeks postnatal) and histology-matched (control Q, 39 weeks in utero) controls showed increased diffuse and lateral expression of the intercalated disc (white arrows). A total of 6–8 images were captured based on 4 independent tissue slice stainings per individual; representative images are shown. The scale bar represents 50 µM.

Additional stainings identifying mechanical components of the ICD (N-CAD and JUP) were performed. N-CAD labeling in control R shows mainly complete and normal-formed ICD structures, with scarce labeling found at the lateral side of cardiomyocytes (Figure 2). Also, a consistent overlap between N-CAD, JUP, and β-catenin was seen, demonstrating the intertwined trafficking processes of these ICD proteins (Figure S1). Whereas patient S presented seemingly decreased protein levels and an absence of ICD formation, patient T showed several appropriate ICDs but also much labeling in a diffuse pattern. Again, these patterns are more comparable to control Q. Overlap between both of the ICD proteins remained similar, as seen in both controls. These results highlight the differences between both patients and the controls, resembling more premature maturation patterns of both the ICD and cardiomyocyte proliferation, with patient T being two weeks older and showing more advanced ICD expression and structure compared to patient S.

**Figure 3:**
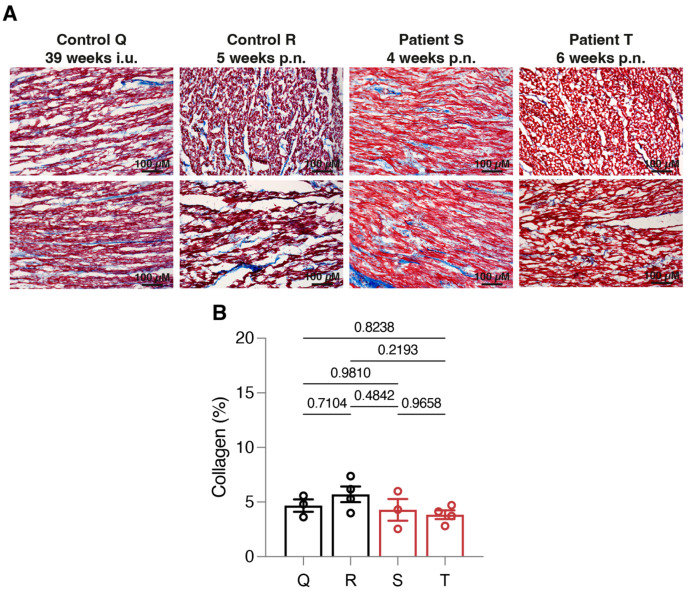
Limited fibrosis formation in pediatric patient tissues. (**A**) Masson’s trichrome staining of myocardial tissues. (**B**) A quantitative comparison of two pediatric patients (patient S, 4 weeks postnatal; patient T, 6 weeks postnatal) to age-matched (control R, 5 weeks postnatal) and histology-matched (control Q, 39 weeks in utero) controls did not show overt collagen deposition (depicted in blue). A total of 3–4 images were captured based on 2 independent tissue slice stainings per individual; representative images are shown. The scale bar represents 100 µM.

Finally, to exclude the involvement of increased fibrosis formation in these patients, Masson’s trichrome stainings were performed (Figure 3). All individuals showed only a mild amount of collagen deposition, suggesting a limited contribution of fibrosis to the enlargement of these pediatric hearts.

## 4. Discussion

During postnatal cardiac maturation, the cardiomyocytes lose the ability to proliferate, and muscle mass is almost entirely increased by hypertrophy of existing cardiomyocytes [9,11,16]. Simultaneously, the protein network of the ICD and the sarcomeres is formed in stages that can be identified by diffuse (unorganized) intracellular protein localization, followed by lateral expression of proteins designated to form the ICD, and eventually resulting in the formation of a clear ICD at the longitudinal contacts [18].

Interestingly, N-CAD is presumed to be one of the first proteins to be trafficked and anchored at the forming ICD, followed by the transportation of other junctional proteins [17,18]. Furthermore, ion channels and connexins (composing gap junctions) that are also part of the area composite will follow afterwards and are therefore affected in situations of N-CAD mislocalization or absence [19]. Also, part of this area composita are catenin proteins, such as JUP (gamma-catenin), α-catenin, and β-catenin, that mainly anchor the cytoskeleton to the ICD thereby providing mechanical strength to the myocardium. Interestingly, both α- and β-catenin are involved in signaling pathways that regulate cardiomyocyte proliferation. Destabilization of the ICD proteins can result in aggregation for α-catenin, or in translocation for β-catenin. Loss of α-catenin results in increased YAP protein accumulation in the nucleus, initiating cell proliferation, as seen during inactive Hippo signaling [16,20]. Similarly, when β-catenin is not sequestered at the area composita due to disturbed N-CAD binding, it translocates into the nucleus as part of the Wnt-signaling pathway, also leading to cell proliferation [21]. While we did not observe clear nuclear β-catenin labeling, protein localization was also disturbed, similarly to JUP and N-CAD. In cases of inappropriate N-CAD localization and ICD formation, these overlapping mechanisms stimulate cardiomyocyte proliferation, leading to hyperplastic rather than hypertrophic development of the myocardium.

The consequences are weakened cell-to-cell conduction of mechanical force propagation and altered electrical activity patterns in the myocardium. This can directly be translated to increased arrhythmogenicity, as that could result in the occurrence of conduction blocks or premature reactivation during cardiac repolarization, leading to reentry or ectopic activity, respectively. The importance of proper ICD formation is accentuated by the incidence of a specific disease named arrhythmogenic cardiomyopathy (ACM), a life-threatening disease that involves mutations in genes encoding major desmosomal proteins in more than half of the patient population [22]. ACM is specifically known for the high occurrence of sudden cardiac death (SCD). Unfortunately, the ACM phenotypes vary between patients and show incomplete penetrance. As proper localization of most of the desmosomal proteins is preceded by proper localization of N-CAD, decreased N-CAD expression could result in a phenotype reminiscent of ACM. This has recently been studied in a subset of ACM patients, showing a comparable N-CAD mutation in genotype-negative ACM patients [23,24], which has previously also been shown for α-catenin [25].

In the presented patient cases, we observed and quantified a vast increase in nuclei counts, as well as increased Ki67-positive nuclei, compared to the age-matched control R. This suggests cardiomyocyte hyperplasia equivalent to control Q tissue at 39 weeks gestation, in which the switch towards physiological hypertrophy has yet to occur. Likely, this shift has not taken place in the patients, resulting in persistent hyperplasia. This manifested in increased heart sizes, which could precipitously be identified as hypertrophy. Simultaneously, the formation of the ICD was not comparable to the age-matched control R, but rather to earlier gestation phases, such as control Q, indicating a delayed cardiac development in the patients. The difference between both patients consisted mainly of the seemingly increased expression and localization of N-CAD in patient T. This is at least partially caused by the older age of the patient, as two weeks of cardiac development can include major changes in terms of ICD formation [18]. Due to N-CAD’s leading role in ICD formation and structuring, the area composita of both patients was still immature. Hyperplastic cardiomyocytes lack the properties of well-organized sarcomere structures as seen in mature cardiomyocytes [26], consequently suggesting a limiting contractile function of these pediatric hearts. Additionally, fragmented formation of the ICD includes dysfunction of ion channels and gap junctions [7,18], which could fuel worsened electrical conduction and increase susceptibility to arrhythmias in these patients.

While it is not a certainty that our two patients suffered from cardiac arrhythmias, the aberrancies regarding the proteins involved in mechanical coupling cannot be disregarded. As genetic mutations in these proteins are linked to life-threatening symptoms, it would be advisable to monitor related and future family members. With future pediatric sudden death cases demonstrating an enlarged heart similar to our cases, it could be medically worthwhile to conduct more in-depth studies in order to reveal more details about the underlying etiology.

## 5. Conclusions

In conclusion, we described two cases in which pediatric patients died within the first 6 weeks after birth. The direct cause of asphyxia remains unclear in these patients, although tissue analysis dictates a leading role for the underdeveloped heart. While the morphological adaptation of the hearts is not comparable with that of ACM patients, potentially due to the very early onset of molecular remodeling, the overlap between alterations of the underlying ICD protein mechanics in ACM and those in these patients remains striking.

## Data Availability

The data presented in this study are available on request from the corresponding author.

## Supplementary Materials

The following supporting information can be downloaded at: https://www.mdpi.com/article/10.3390/jcdd10080354/s1, Figure S1: Immature intercalated disc maturation in pediatric tissues. Immunohistochemical labeling of N-cadherin (N-CAD) and nuclei (DAPI) in combination with either plakoglobin (JUP, **A**) or β-catenin (**B**), at 60×. Intercalated disc proteins JUP and β-catenin are expressed in similar patterns as N-CAD. The two pediatric patients (patient S, 4 weeks postnatal; patient T, 6 weeks postnatal) when compared to age-matched (control R, 5 weeks postnatal) and histology-matched (control Q, 39 weeks in utero) controls showed increased diffuse and lateral expression of the intercalated disc, alongside several completely formed intercalated discs (white arrows). Four images were captured based on four independent tissue slice stainings per individual; representative images are shown. The scale bar represents 50 µM.
