# Supplementary material for: Maturation and Function of the Intercalated Disc: Report of Two Pediatric Cases Focusing on Cardiac Development and Myocardial Hyperplasia
Source: J Cardiovasc Dev Dis. 2023 Aug 19;10(8):354. doi: 10.3390/jcdd10080354 (PMC10455643; doi:10.3390/jcdd10080354)
Supplement: Supplementary file 1 [file jcdd-10-00354-s001.zip › jcdd-2565894-supplementary.pdf]

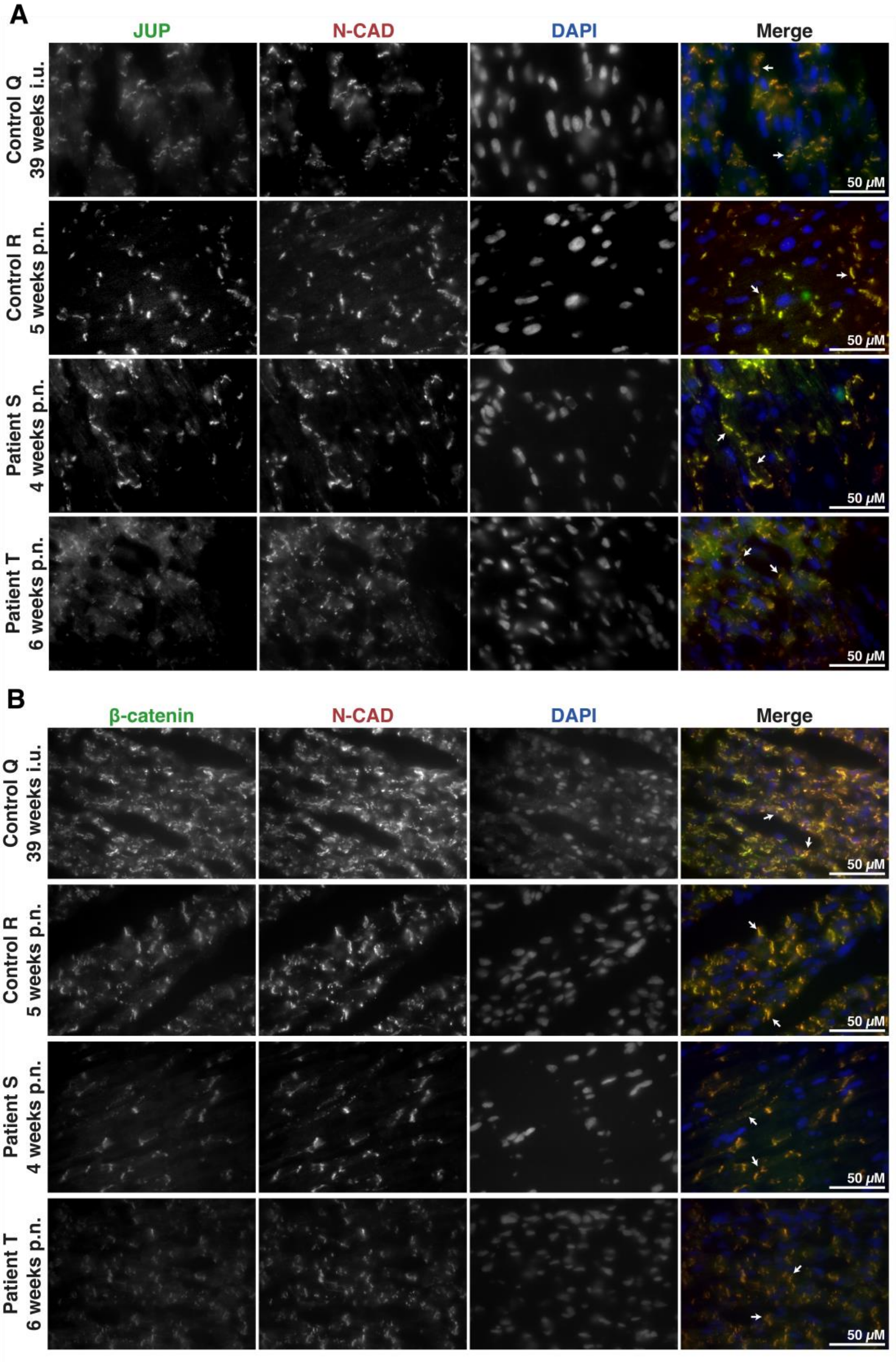

Figure S1. Immature intercalated disc maturation in pediatric tissues. Immunohistochemical labeling of N-cadherin (N-CAD) and nuclei (DAPI) in combination with either plakoglobin (JUP, **A**) or β-catenin (**B**), at 60x. Intercalated disc proteins JUP and β-catenin are expressed in similar patterns as N-CAD. The two pediatric patients (patient S, 4 weeks postnatal; patient T, 6 weeks postnatal) when compared to age-matched (control R, 5 weeks postnatal) and histology-matched (control Q, 39 weeks in utero) controls showed increased diffuse and lateral expression of the intercalated disc, alongside several completely formed intercalated discs (white

arrows). 4 images were captured based on 4 indepent tissue slice stainings per individual, representative images are shown. Scale bar represents 50 μM.
